# SEIR model and simulation research on unsafe psychological state propagation of construction workers considering safety climate and intimate relationships

**DOI:** 10.3389/fpubh.2022.1031440

**Published:** 2022-10-12

**Authors:** Ruijia Yuan, Zhiwei Zhang, Xiaopeng Deng, Xiaosheng Li

**Affiliations:** ^1^School of Management Science and Engineering, Anhui University of Finance and Economics, Bengbu, China; ^2^School of Civil Engineering, Southeast University, Nanjing, China; ^3^School of Statistics and Applied Mathematics, Anhui University of Finance and Economics, Bengbu, China

**Keywords:** safety climate, intimate relationships, unsafe psychology, SEIR model, simulation research

## Abstract

The construction industry is a pillar industry of China and occupies an essential position in our economic development. However, in the fast-developing construction industry, the number of its safety accidents is also growing year by year. Safety accidents are often due to unsafe behaviors of construction workers, and unsafe precarious psychological states are important factors for unsafe behaviors. Therefore, this paper, based on a review of existing literature, uses the SEIR model and numerical simulation method to study the spread of unsafe psychological states among construction workers considering safety climate and intimate relationships. It puts forward corresponding countermeasures, which has great practical significance for reducing safety accidents in the construction industry. The results show that: (1) A good safety climate can help alleviate the spread of unsafe psychological states of construction workers. (2) The intimate relationship between construction workers will promote the association between communicable people and susceptible people, which will lead to the spread of an unsafe psychological state. (3) A larger network average degree will increase the spread speed and the density of communicable people, but will not increase the spread range.(4) Forgetting rate has a key role in the propagation of unsafe psychological states. Suggestions are made to hinder the propagation of these states, which will help to reduce the unsafe behavior of construction workers and the accident rate in the construction industry.

## Introduction

The construction industry is an essential force driving the national economy and is a key influence in the development of the country. For a country with a large population like China, the status of the construction industry is more prominent than that of other countries. In recent decades, Chinese authorities have making efforts to push forward the growth of the construction industry by promoting the transformation and upgrade of the construction industry toward intelligence, industrialization and informationization. In May 2022, the Ministry of Housing and Urban-Rural Development of the People's Republic of China (MOHURD) published the “Notice on Soliciting and Selecting Intelligent Construction Pilot Cities” (1), with the aim of playing a demonstrated and leading role in comprehensively promoting the transformation and upgrade of the construction industry and driving high-quality development. The same ministry issued the “ '14th Five-Year Plan' for the development of the construction industry” (2). The reform and development of China's construction industry during the “13th 5-Year Plan” has been effective, with an average annual growth of 5.1% added to the value of the national construction industry, which accounts for more than 6.9% of the GDP. The role of the construction industry as a pillar industry of the national economy continues to grow, and this promotes economic growth, alleviates the pressure of difficult employment, promotes the development of a new type of urbanization to protect and improve people's lives within a moderately prosperous society. However, while a series of achievements have been made, its quality and efficiency problems in the industry remain, and safety accidents during construction are frequent. According to the notification of production safety accidents in housing and municipal engineering released by the MOHURD in 2019 (3), there were 773 production safety incidents and 904 deaths in housing and municipal engineering nationwide in 2019, 39 more accidents and 64 more deaths than in 2018, up 5.31 and 7.62%, respectively. Among these there were 23 large and major safety accidents, with 107 fatalities, an increase of 1 accident and 20 fatalities over 2018, up 4.55 and 22.99%, respectively. These data show that the construction industry in China is high risk and has a high accident rate, which obviously runs counter to the safety development concept of China's construction industry.

According to the accident cause theory, accidents can be attributed to the unsafe behaviors of workers and the unsafe state of objects (4). Unsafe behaviors of workers include disobeying orders, rule-breaking operations and human operating errors. The unsafe state of objects includes inadequate or defective safety protection equipment, performing construction activities in bad weather or with insufficient lighting conditions (5). People are the main body of construction activities, and unsafe human behavior is the most important factor that affects construction safety (6); 90% of accidents at construction sites are caused by human errors, and 88% of construction accidents involve human unsafe behavior. The main reason for the high incidence of construction accidents in China lies in the unsafe behaviors of construction workers; however, the unsafe behaviors are the external manifestations of workers, and the most important determinant of unsafe behavior lies in human psychology (7), and most of unsafe behavior is directly caused by people's unsafe psychology. The classic unsafe psychology include fluke psychology (believing that danger cannot happen to themselves), energy-saving psychology (willingness to take risks to save time or energy), paralysis psychology (inability to make good decisions quickly when necessary), herd psychology (seeing everyone doing it so they do it too), rebellious psychology (not accepting the right advice and insisting on wrong behavior), etc. These psychologies make workers pay less attention to safety, thus ignoring the objective safety risks and making unsafe behaviors that endanger life and health. These psychologies are also affected by many external factors, such as physical condition, fatigue, labor intensity, etc. The study of worker's psychological activities can effectively control human behaviors, and improving the psychological state of workers is one of the most effective ways to reduce the risk of unsafe behavior (8), which can greatly reduce the probability of accidents and thus ensure production safety. And according to social network theory, construction workers form a complex network with each other as a group because of relational ties, and the network can serve as the main path for the spread of behaviors (9). Meanwhile, the unsafe psychological state can also spread through this relational network, and if it left unchecked to greatly increase the probability of safety accidents. Therefore, it is worthwhile to think about how these unsafe psychologies spread and how to reduce or even block their spread. When it comes to the propagation of psychology, it is obvious to easily spread among people who have intimate relationships, and for safety issues in the construction industry, “safety climate” is a common leading indicator. Therefore, this paper addresses the current situation of frequent accidents in the construction industry and takes the propagation of unsafe psychological states of construction workers as a research content in order to reduce the occurrence of accidents on construction sites, and innovatively applies the SEIR model to the construction field as well as taking both safety climate and intimacy into consideration as a way to analyze the propagation of unsafe psychological states. Suggestions are made to hinder the propagation of these states, which will help to reduce the unsafe behavior of construction workers and the accident rate in the construction industry. These suggestions will of great significance in achieving a high-quality low-accident construction industry in the new era.

## Literature review

### Safety climate

In 1980, the Israeli scholar Zohar was the first to propose the concept of “safety climate.” He pointed out that safety climate is a common perception of members in an organization about safety issues and working environment (10). This common perception is influenced by various factors within the organization, such as safety training, safety communication, safety systems, safety awareness of employees. Together these safety-related factors form a safety climate and thus affect the safety behavior of employees. Scholars' research on safety climate is mainly divided into two types.

The first type is the research on the safety climate model. Based on an artificial neural network (ANN) using a three-layer feed-forward back propagation neural network to analyze the data, Patel developed a model that can reasonably predict the safety climate of construction projects (11). Chen introduced the concept of elasticity into the safety climate model, and developed and verified the Safety ClimateResilience (SCR) measurement model. SCR is measured by seven dimensions: management commitment, supervisor safety perception, colleague safety perception, learning, reporting, expectation and awareness (12). Probst developed and verified a new safety climate assessment tool (S-CAT), which contains 37 independent indicators of eight safety climate elements determined by construction industry specialists, and is used to self-assess the maturity of the organization's safety climate (13).

The second type is to research the relationship between safety climate and safety behavior, safety results, safety performance, safety knowledge, etc. Bhavana evaluated the influence of safety climate on hazard identification and safety risk recognition level. The research shows that safety climate has a positive effect on hazard identification and safety risk identification level, and safety climate has an indirect impact on safety risk identification through hazard identification (14). He studied the relationship between safety climate, safety behaviors and safety outcomes between supervisors and construction workers. The results show that there is a positive association between safety climate and safety behaviors of the two groups, but there are significant differences between safety climate and safety outcomes, safety behaviors and safety outcomes (15). Borgheipour studied the moderating effect of safety knowledge and the mediating effect of safety motivation, discussed the relationship between safety climate and safety performance in the cement industry, and proved that a good safety climate promotes safety motivation and safety knowledge that reduces unsafety behaviors (16). Chen studied the relationship between safety climate and the safety behaviors of construction workers in Taiwan Province, and found that there is a significant positive correlation between safety climate and safety behavior. The level of safety climate has a demonstrable impact on safety behavior and overall safety perception, and the dimension of “safety promise and safety training” has the most significant impact within a safety climate (17).

### Intimate relationships

Relationships are often seen as personal or inter-organizational ties formed through high-quality social actions and mutual exchange of interests (18). Relationships may be between peoples (e.g., interpersonal relationships) or between groups (e.g., inter-organizational relationships). From the connotation of interpersonal relationship, King pointed out that an interpersonal relationship under the background of Chinese traditional culture is formed based on the exchange and sharing of social experience among individuals, and may come from relationships with genealogical relatives, the neighborhood, classmates, colleagues, teachers and students, superiors and subordinates, or friends with the same hobbies (19). Based on its closeness, an interpersonal relationship can be divided into intimate or non-intimate. “Intimacy” can be characterized as broad or narrow (In this paper, “intimacy” has the same meaning as “intimate relationship”). The narrow meaning of intimacy refers to marriage or a loving relationship between individuals, while the broad meaning of intimacy is related to the degree of dependence between two people, who can be friends, fellow villagers, classmates, relatives, and so on. Based on these definitions, this paper combines the characteristics of social relations of construction workers and uses the broad sense of intimacy for analysis. Yagil & Luria concluded that high-quality intimacy interacts with employees' perceptions of climate to compensate for the effects of low levels of safety climate (20). Liu, based on the social exchange theory, studied the mechanism of workplace deviant behavior (WDB) so as to offer a theoretical rationale for its precautionary management. The results show that Leader-member exchange (LMX) and Leader-member guanxi (LMG) have direct mitigative effects on WDB, and LMG plays a regulatory role in this process. Therefore, decreasing or eradicating WDB by construction workers may depend not just on formal controls, but also on building good LMX and LMG (21).

### Unsafe psychology

“Unsafe psychology” refers to workers working under the influence of various degrees of stress, physical health, fatigue, safety climate, safety training and other factors, into a psychological state consisting of anxiety, depression, irritability and other moods intertwined. These factors can make workers be inattentive toward safety risks, which leads to unsafe behavior, and thus is a personal safety factor. According to the literature, scholars' research on unsafe psychology has mainly focused on exploring the relationship between unsafe behaviors and unsafe psychology, and provides suggestions for reducing unsafe behavior. Li studied the influence between the tendency to indulge in unsafe behavior and conflict management strategies through structural equation model. The research showed that unsafe psychology mediates the relationship between management strategies and coal miners' tendency to behave unsafely, and that to some extent interpersonal intimacy plays a moderating effect between the three conflict management strategies and unsafe psychology (22). Yu studied the use of the Behavior-based Safety management (BBS) approach in the coal mining industry and contrasted miners' unsafe behavior and psychology of miners before and after the application of BBS, and the results showed that the implementation of BBS was effective in reducing the accident rate of coal miners, and that unsafe behavior of miners were closely related to psychology (23). Tong constructed JD-R model to study miners' unsafe behavior, considered the role of psychological factors, and proposed dual-process management from the perspective of organization-occupational psychology-behavior to reduce miners' unsafe behavior (8). Li analyzed the influential factors in the cognitive process of construction workers from the perspective of safety cognition based on multi-agent modeling, constructed interaction and perception. The study set behavioral rules and parameters for simulation under the two-way effect of formal rule awareness and herd mentality model, and the results show that workers' psychology and consciousness influence the perception process, and the higher the level of herd psychology, the more likely are unsafe behaviors by the group (6).

### SEIR model

The SEIR model is a new infectious disease model proposed by Anderson and May in 1991 based on the SIR model with the addition of ‘exposed,’ The letters of the acronym refer to Susceptible, Exposed, Infectious and Recovered, respectively. SEIR model is often applied to analyze and forecast the propagation of epidemic diseases, such as HIV, Ebola virus, SARS virus, and in recent years, SEIR model has made great achievements in the context of the COVID-19 epidemic, and some scholars have applied SEIR model to other fields. Yuan proposed an E-SIER model to explore virus spread in networks with P2G information sharing mode, and studied the short-term outbreak and long-term network viability of network viruses (24). Liu considered the probability that exposed nodes may be removed, and a SEIR model of rumor propagation on heterogeneous networks was proposed. In accordance with the model, two immune strategies of rumor propagation are discussed, and numerical simulation proves their effectiveness (25). Sachak-Patwa found that video virus propagation is similar to infectious disease propagation, formulated a SEIR model including time delays to describe the epidemic evolution of virus-infected videos, and verified the model by fitting the model parameters to data on watching YouTube music videos (26).

Based on the above research, and inspired by the spread of unsafe psychological states within construction workers, this paper applies the SEIR model to the construction field to study the propagation of unsafe psychological states in groups of construction workers. It takes safety climate (the leading index that has been proved to be related to safety problems) and intimacy (the index that may be related to spread) into account, and verifies the model by numerical simulation and puts forward some suggestions.

## Problem description and hypotheses

### Problem description

Unsafe psychology is the direct cause of dangerous and unsafe behavior (22), and unsafe behavior creates conditions that results in accidents (8). It was found in the field survey that during the project construction process, when construction workers come into contact with people having an unsafe psychological state, some workers will learn, imitate, spread the unsafe state; others will not be affected by the unsafe psychological state of the infectors around them because of their own strong safety awareness or organizational safety climate. Based on this, according to the characteristics of the spread of construction workers' unsafe psychological state, and with reference to Sachak-Patwa's criteria for the division of Internet user groups in his research on the propagation of viral videos (26) and Liu's criteria for dividing groups of Weibo users in the study of rumor spread on heterogeneous networks (27), the construction workers can be divided into four categories:

S: Susceptible, people who have no existing unsafe psychological state but are very likely to be affected by the unsafe psychological state of construction workers around them;
E: Exposed, the potential communicators who have come into contact with construction workers with unsafe psychological state, without the ability to propagate;
I: Infectious, construction workers with unsafe psychological state and may propagate the state;
R: Recovered, people who have strong safety awareness or are constrained by external environment such as safety climate and safety supervision can fully resist being influenced by communicators with unsafe psychology; however, immunity period is limited.

### Basic hypotheses

**Figure 1:**
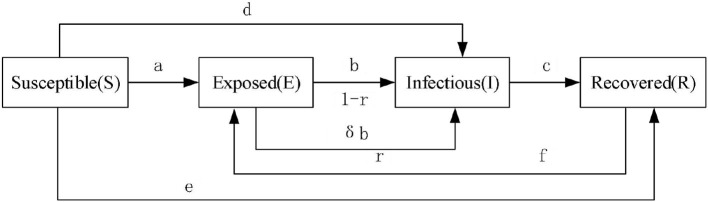
Conceptual model of propagation process of unsafe psychological state of construction workers.

Based on the above problem description, a conceptual model of the propagation process of construction workers' unsafe psychological state, considering safety climate and intimacy, is constructed, as shown in Figure 1, and the following basic hypotheses are put forward:

(1) Acording to the assumption of constant number of in Sachak-Patwa's study population (26), and constant number of microblog users in Liu's study (27), so this paper assumes that in the process of spreading unsafe psychological state, the research object is construction workers in an area, and the exit and entry of construction workers in this area are not considered; that is, the number of workers is constant;
(2) Based on the social network theory, construction workers can be considered as nodes and the relationship between them as edges, we assume that the spread of construction workers' unsafe psychological state has X nodes and Y edges, and each node has K adjacent nodes (25);
(3) Susceptible persons are easily influenced by construction workers with unsafe psychological state. Generally, they become exposed with probability a, and a is constant. There is probability d that susceptible persons will become infectious persons with unsafe psychological state, where d is the direct infection rate. In addition, under the influence of safety climate, they may also change from susceptible persons to recovered persons with probability e, where e is the level of environmental safety climate;
(4) Exposed persons come into contact with non-intimate infectors with probability r, and become infectors with probability δb. They come into contact with intimate infectors with probability 1-r, and become infectors with probability b, where b is infection rate;
(5) After a period of propagation, the infectors with unsafe psychological state will change into a recovered person with probability c (28), where c is the improvement rate. (This may be due the infectious worker being warned about site accidents being caused by unsafe psychological state, or by being supervised.
(6) Considering that the recovered state of construction workers' unsafety psychology is a temporary immunity (28), recovered persons will become susceptible due to forgetfulness or excessive work stress with probability f.

## Model building and analysis

### Model building

The unsafe psychological state of construction workers is often the main source of accidents on construction sites. For the purpose of analyzing the impact mechanism of construction workers' unsafe psychological state, based on problem description and hypothesis, a SEIR model of unsafe psychological state propagation of construction workers considering safety climate and intimacy was constructed. This model is a uniform network propagation model with approximately equal node degree. The total number of construction workers group in a certain area is T, the number of relationships among them is W, and K is the average degree of network. S (t), E (t), I (t) and R (t) are used to denote the density of susceptible persons, exposed persons, infectors and recovered persons corresponding to time *t*, respectively. These constitute the total number of construction workers in a construction project within a certain area and satisfy the normalization condition. Therefore, at any time.


(1) $$\begin{array}{l} S_{t}+E_{t}+I_{t}+R_{t}=1 \end{array}$$

**Figure 2:**
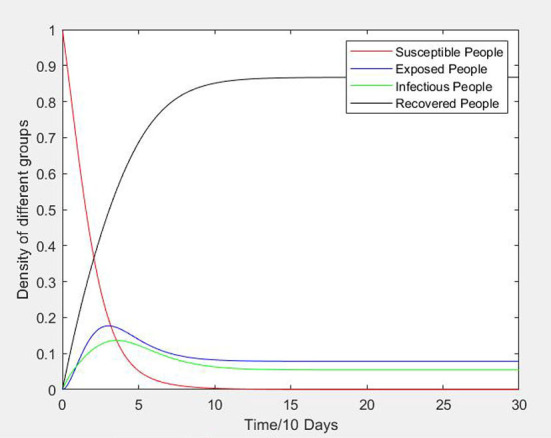
Variation curve of node density with time.

Based on the propagation process of construction workers' unsafe psychology illustrated in Figure 2 below, combined with the above basic hypotheses and the principle of propagation dynamics, a differential dynamics model for propagating SEIR unsafe psychological states of construction workers considering safety climate and intimacy is established:


(2) $$\begin{array}{r} \frac{dS_{t}}{dt}=-aKS_{t}I_{t}-dS_{t}-eS_{t} \end{array}$$

(3) $$\begin{array}{r} \frac{dE_{t}}{dt}=aKS_{t}I_{t}-rdbE_{t}-\left(1-r\right)bE_{t}+fR_{t} \end{array}$$

(4) $$\begin{array}{r} \frac{dI_{t}}{dt}=dS_{t}+rdbE_{t}+\left(1-r\right)bE_{t}-cI_{t} \end{array}$$

(5) $$\begin{array}{r} \frac{dR_{t}}{dt}=eS_{t}+cI_{t}-fR_{t} \end{array}$$

### Steady-state analysis of model

The differential dynamic model for propagating SEIR of construction workers' unsafe psychological state considering safety climate and intimacy describes the density change and interdependence of four kinds of nodes in a uniform network. Formula (2) is the density change rate of susceptible persons at T time, formula (3) is the density change rate of exposed persons at T time, formula (4) is the density change rate of infectors at T time, and formula (5) is the density change rate of recovered persons at T time. With the passage of propagation time, the density of the four types of nodes changes continuously until the system is in a dynamic equilibrium state and the infection process ends.

In order to further analyze the steady state of the model, formulas (2) to (5) are all equal to zero, which means that the density change rates of the four types of nodes are all zero, and the system is in a state of dynamic equilibrium. Thus, the steady state equations of the model are constructed as follows (6).


(6) $$\{\begin{array}{c} -aKS_{t}I_{t}-dS_{t}-eS_{t}=0\\ aKS_{t}I_{t}-rdbE_{t}-(1-r)bE_{t}+fR_{t}=0\\ dS_{t}+rdbE_{t}+(1-r)bE_{t}-cI_{t}=0\\ eS_{t}+cI_{t}-fR_{t}=0 \end{array}$$

After calculation are performed, we can see that the stable point St has two values (0 and S^*^t). When the value of St is not equal to zero but S^*^t, the stable point I^*^t = (-d-e)/aK; that is, I^*^t < 0. Obviously, a stable point I^*^t of less than zero has no practical significance, so St = 0. Then, St = 0 is substituted into formulas (1) and (6), and the equations are combined to solve the stable points as follows (7).


(7) $$\{\begin{array}{c} S_{t}^{*}=0\\ E_{t}^{*}=\frac{cf}{cf+(c+f)(rdb+b-rb)}\\ I_{t}^{*}=\frac{f(rdb+b-rb)}{cf+(c+f)(rdb+b-rb)}\\ R_{t}^{*}=\frac{c(rdb+b-rb)}{cf+(c+f)(rdb+b-rb)} \end{array}$$

## Numerical simulations and discussion

Based on a field investigation of front-line construction workers in Yangtze River Delta region, it is known through interviews that construction workers mainly work as construction teams such as steel bar, concrete, formwork and masonry teams. According to the project scale, each team has 5–20 people, and an unsafe psychological state of construction workers in a team will influence other workers within the team. Computer software is used to simulate the mathematical model. According to the propagation rules of an unsafe psychological state in social network discussed above, and through field investigations and expert interviews, the simulation parameters are set as follows: *N* = 15; When t = 0, S(0) = 1, E(0) = 0, I(0) = 0, R(0) = 0, K = 4, a = 0.7, b = 0.7, c = 0.8, d = 0.1, e = 0.2, f = 0.05, r = 0.3, δ = 0.3.

### Variation of node density with time

As shown in Figure 2, the basic curves describe the propagation and evolution of an unsafe psychological state of construction workers in a uniform network. The density of susceptible persons decreases and tends to become stable with the passage of time, which indicates that the unsafe psychological state of construction workers spreads rapidly in the social network of construction teams and groups, and occurs mainly in the first 10 days when people are in contact with each other. The density curves of exposed persons and infectors are similar, and increase rapidly in the initial stage but then gradually decrease after reaching the peak until they reach a stable state, Recovered persons gradually increase in the early propagation stage of an unsafe psychological state of construction workers, continue to increase before gradually reaching a stable state at a later stage.

This process is consistent with reality, where an unsafe psychological state can be spread via the social network relationships of construction workers. At the initial stage of the spread of an unsafe psychological state, due to the lack of sufficient understanding of the construction industry or the influence of an unsafe psychological state of workers in the team, construction workers unconsciously express their unsafe psychology as luck, energy or time saving and paralysis in the construction process. As the unsafe psychology state is absorbed, accepted and spread, the density of infectors reaches the maximum. After that, with safety supervision or warnings of construction site safety accidents caused by unsafe psychological states, or an understanding of the negative impacts of an unsafe psychological state, construction workers improve their own safety awareness. Therefore, they lose interest in propagation, slow down the propagation rate, and largely stop spreading the unsafe psychological state. These workers then become recovered. However, some workers are at risk of becoming exposed workers due to forgetting or excessive work pressure.

### Influence of safety climate and intimacy on node density

#### Safety climate

**Figure 3:**
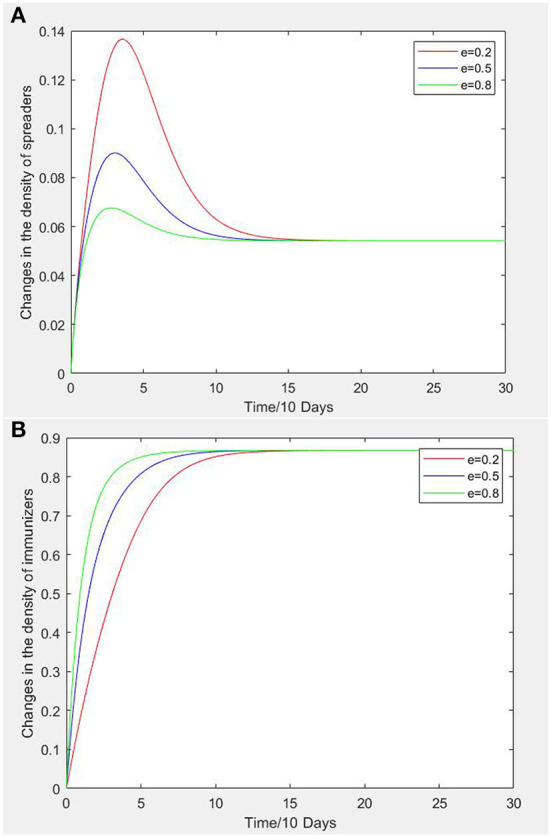
**(A)** Variation curves of infectors density under different safety climate intensity. **(B)** Variation curves of recovered persons density under different safety climate intensity.

After Zohar put forward the concept of safety climate for the first time and defined it as “the consensus of all members of the organization on a safe working environment,” it has aroused widespread interest among scholars. Scholars generally believe that safety climate is a kind of employees' perception of safety-related issues. The safety climate of construction team emphasizes that workers have a common perception of safety-related problems emanating from the working environment of construction team, team leader and team members when completing the established construction tasks as a team. According to the dynamic equation analysis of the SEIR propagation model of construction workers' unsafe psychological state, it can be seen that safety climate e has distinct influence on the propagation of an unsafe psychological state. From field investigation data, the safety climate e are set at 0.2, 0.5, and 0.8 for data simulation analysis. Figures 3A,B show the changes of infectors and recovered persons density with time when e is taken as 0.2, 0.5 and 0.8. In Figure 3A, when e is 0.2, the peak time of infectors density curve is slightly slower than that of e is 0.5 and 0.8, but the peak value is more than twice as high as e = 0.8. In the recovered density curve of Figure 3B, the growth rate of the curve is faster when e is 0.8 than when e is 0.2 and 0.5.

Through the analysis of Figures 3A,B and as reflected in actual situations, it is not difficult to see that the safety climate of teams and groups has an important influence on the unsafe psychological state of construction workers. The greater the level of safety climate, the more it helps to inhibit or restrict infection and the spread of unsafe psychological states, so susceptible persons are not affected by an unsafe psychological state, or can become recovered persons possessing safety awareness. Therefore, the construction unit should create a safety climate that continuously increases the education of workers regarding construction accidents, improves the safety awareness of the team members, and improves the overall safety climate level of the team.

#### Intimate relationships

**Figure 4:**
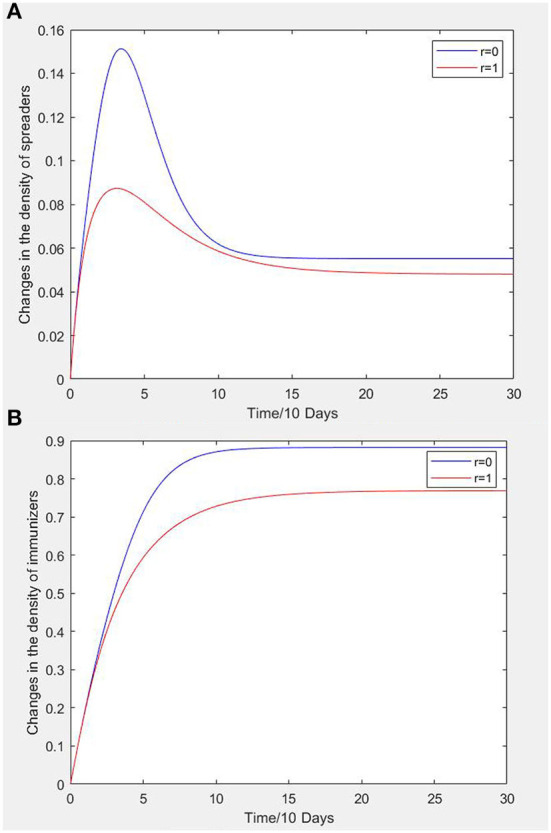
**(A)** Effect of the presence or absence of intimacy on changes in infectors density. **(B)** Effect of the presence or absence of intimacy on changes in recovered persons density.

Under the condition that other parameters are unchanged, the influence of intimacy on node density is analyzed. When *r* = 0, all susceptible persons become exposed persons by contacting intimate infectors with an unsafe psychological state. When *r* = 1, all susceptible persons become exposed persons by contacting non-intimate infectors with an unsafe psychological state. In the two cases, the density curves of infectors and recovered persons with time is shown in Figure 4. It can be seen that the density curves of infectors and recovered persons show the same trend with time irrespective intimacy, but range is different to some extent.

It can be seen from Figure 4A that when *r* = 0, the change amplitude and peak value of infectors density curve is larger than when *r* = 1. Intimacy enhances the imitation and propagation effect of the exposed persons, thus increasing the number of infectors.

From Figure 4B, it can be seen that when *r* = 1, the density increase of recovered persons is smaller than that when *r* = 0, and the systems both tend to be stable at about *t* = 12 days. This is due to the fact that the density of stable points of infectors under intimate relationship is higher than that under non-intimate relationship. When the *r* = 0, the density of stable points of recovered persons is obviously higher than *r* = 1.

It is assumed that the peak density of infectors represents the ability of construction workers' in an unsafe psychological state to spread this state; that is, when the system is in steady state, the greater the density of propagators of the unsafe psychological state, the greater the propagation ability of workers in an unsafe psychological state in the system, and the more likely it is to trigger unsafe behavior and even cause accidents.

The density of recovered persons represents the speed of spreading an unsafe psychological state amongst construction workers; that is, when the system is in steady state, the smaller the density of recovered persons, which indicates that the unsafe psychological state spreads faster in the system and causes construction safety accidents faster. The simulation results show that the ability and rate of propagation of unsafe psychological states of construction workers with non-intimate relationships in construction teams are low, which is consistent with reality. When the infectors with an unsafe psychological state and the exposed persons are in a non-intimate relationship, the exposed persons will become the infectors of the unsafe psychological state of construction workers with a probability δb, which is smaller than the transition probability b if an intimate relationship existed between them. Therefore, in the social network formed by construction workers, we should concentrate on construction workers with intimate relationships because this will be an important way to slow down spread of the unsafe states.

### Influence of network average degree K and forgetting rate f on node density

#### The effect of network average degree on the density of each node

**Figure 5:**
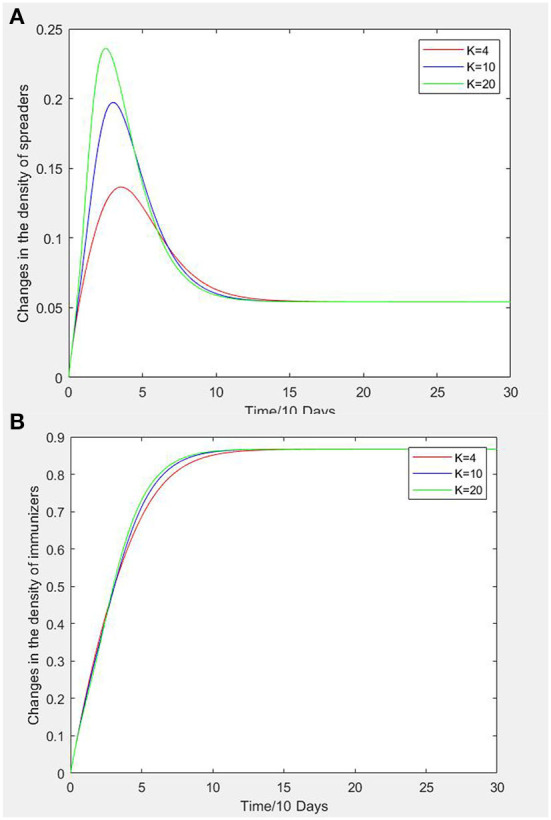
**(A)** Variation curves of network average degree vs. infectors density. **(B)** Variation curves of network average degree vs. recovered persons density.

In a social network, the degree of a node is the number of edges adjacent to it, and the average degree K of the network refers to the average of the sum of the degrees of all nodes in the social network, which can represent the density of the social network. From the dynamic equation of the SEIR propagation model in a uniform network, it can be found that K has certain influence on the propagation of an unsafe psychological state. Under the condition of keeping other parameters unchanged, numerical simulation experiments are carried out K taking 4, 10, and 20. Figures 5A,B shows the variation of transmitter and recovered persons density with time when K is taken as 4, 10, and 20. In Figure 5A, when K is 4, the time of infectors density curve to reach a peak is slower than for K 10 and 20, and the peak value is smaller. In the recovered persons density curve of Figure 5B, when K is 4, the curve grows more slowly than when K is 10 or 20, but the steady value of recovered persons density does not change with K.

Therefore, with the increase of K, the peak value of infectors density curve becomes larger and the peak time appears earlier. The density curve of recovered persons increases faster, and the time to reach the steady state is advanced, but the steady state values are consistent. The results showed that K has an important influence on infectors and recovered persons in the process of spreading an unsafe psychological state of construction workers, but does not change the steady-state value of recovered persons density. This is because with an increase in K, the contact and information exchange among construction workers increases in the unsafe behavior propagation network, which accelerates the spread of unsafe psychological states amongst construction workers and leads to a higher probability of construction safety incidents.

#### The effect of forgetting rate on node density

**Figure 6:**
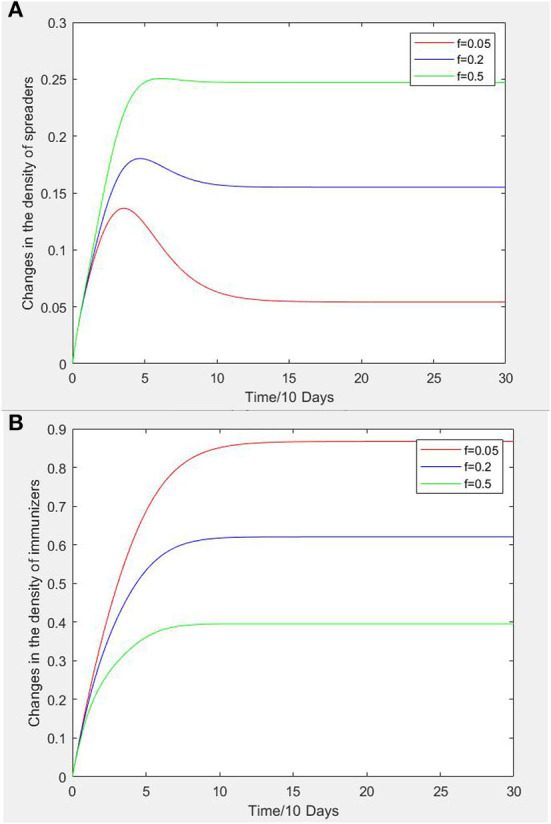
**(A)** Curves of forgetting rate vs. infectors density. **(B)** Curves of forgetting rate vs. density of recovered patients.

Figure 6 shows curves of infectors and recovered persons density changing with time in social network when f has the values of 0.05, 0.3, and 0.7. It can be seen that under the condition that other parameters remain unchanged, as the forgetting rate e increases, the rates of increase to the peak of the three curves are close for a period of time but then diverge, with the greater the forgetting rate e, the higher the peak value of infectors density curve and the lower the peak value of recovered persons density curve.

Forgetting rate e refers to the probability that construction workers will change from recovered persons to exposed persons due to weak memory or excessive pressure. Therefore, forgetting rate e is mainly affected by factors such as inattention and decline of safety awareness. Therefore, construction enterprises can strengthen their safety awareness by regular safety training for employees who have or had unsafe psychological states, and managers can also let model employees play an exemplar role, and enhance the safety climate of construction teams The aim is reduce recovered workers forgetting rate and prevent them from reverting back exposed persons or infectors.

## Conclusions and suggestions

Most safety accidents in construction industry are triggered by unsafe behaviors by construction workers, and the unsafe psychological state of construction workers determines their unsafe behaviors. Therefore, with the help of the SEIR model this paper concentrates on the construction workers' unsafe psychological state, considers factors such as safety climate and intimate relationship, and reveals the spread of unsafe psychological states of construction workers. The paper puts forward the following conclusions and suggestions.

(1) Safety climate helps to alleviate or even restrain the spread of the unsafe psychological states of construction workers, and the density of the spreaders of unsafe psychological states by workers in higher safety climate is low and the density of immune workers is high, which indicates that safety climate plays a “barrier” role in the process of unsafe psychological spread. The dimension of “safety promise and safety trainings” has the most significant impact on the safety climate (17), site managers should hold various safety training meetings on a regular basis. Through the emphasis on safety at the meetings and the guidance of the team leader, the relationships between team members can be enhanced and safety communication promoted. Safety training meetings will also promote understanding and attention to safety among team members, and long-term emphasis will form a construction site safety awareness among team members, thus enhancing a positive safety climate in the construction team as well as improving construction workers' insight into unsafe psychological states. Meanwhile, construction organizations need to monitor employees' mental health, correct workers' unsafe psychological states in a timely manner, and develop training programs focused on improving employees' mental health, especially post-traumatic mental health (12).

(2) Improving psychological states is an effective way to reduce the risk of unsafe behaviors (17), and in the SEIR model of the unsafe psychological state propagation among construction workers, intimate relationship is the basis of trust among construction workers, which promotes effective communication and interaction between transmitters of an unsafe psychological state and susceptible persons. Non-intimate relationships among construction workers to effectively inhibit the ability and speed of unsafe psychological propagation. However, interactions between workers involving high-quality intimate relationships and employees' climate perceptions can compensate for the impact of a low-level safety climate (20). Therefore, team leaders, technical experts, and safety pacesetters should act as key figures who can correct unsafe psychological states of construction workers in a timely manner and can take measures to promote a high level of safety climate with high intimacy.

(3) The network average degree is an important parameter of the network topology of construction workers' unsafe psychological states, and has an important influence on the propagation of unsafe psychological states. A larger network average degree increases the speed of psychological state propagation and the density of transmitters. However, the propagation range will not change. Therefore, according to reinforcement theory, a reward and punishment system can be implemented where construction workers with unsafe behaviors can be punished by negative reinforcement, and construction workers with strong safety awareness and compliance with safety norms can be rewarded, in order to reduce the network average degree of construction workers with unsafe psychological state.

(4) Forgetting rate has a critical role in the propagation of unsafe psychological state. Construction enterprises can increase the pre-employment safety training for construction workers, and strengthen the mutual safety supervision and management system among construction workers. Other measures that can be undertaken include raising group awareness and carrying out psychology education programs so that workers can both take care of each other, remind each other of ways to avoid unsafe work behavior.

## Data availability statement

The original contributions presented in the study are included in the article/supplementary material, further inquiries can be directed to the corresponding author/s.

## Ethics statement

Ethical review and approval was not required for the current study in accordance with the local legislation and institutional requirements. Written informed consent for participation was not required for this study in accordance with the national legislation and the institutional requirements.

## Author contributions

RY and XD conceived the general framework of the article. RY and XL constructed the simulation model and carried out numerical simulations. ZZ edited the manuscript. XD performed the derivation of the equation model. All authors participated in the manuscript review and approved the submitted version.

## Funding

This work was supported by the Anhui University of Finance and Economics research project in 2022 (ACKYC22052), Youth Project of Natural Science Foundation of Anhui Province (2108085QG297), Four New Research and Reform Practice Project of Higher Education Quality Project in Anhui Province (2021sx005), the Teaching Guidance Sub-Committee of Engineering Management and Engineering Cost of the Ministry of Education in 2021 (CMPC202119).

## Conflict of interest

The authors declare that the research was conducted in the absence of any commercial or financial relationships that could be construed as a potential conflict of interest.

## Publisher's note

All claims expressed in this article are solely those of the authors and do not necessarily represent those of their affiliated organizations, or those of the publisher, the editors and the reviewers. Any product that may be evaluated in this article, or claim that may be made by its manufacturer, is not guaranteed or endorsed by the publisher.
